# Gene-edited babies: What went wrong and what could go wrong

**DOI:** 10.1371/journal.pbio.3000224

**Published:** 2019-04-30

**Authors:** Haoyi Wang, Hui Yang

**Affiliations:** 1 State Key Laboratory of Stem Cell and Reproductive Biology, Institute of Zoology, Chinese Academy of Sciences, Beijing, China; 2 Institute for Stem Cell and Regeneration, Chinese Academy of Sciences, Beijing, China; 3 University of Chinese Academy of Sciences, Beijing, China; 4 Institute of Neuroscience, State Key Laboratory of Neuroscience, Key Laboratory of Primate Neurobiology, Shanghai Institutes for Biological Sciences, Chinese Academy of Sciences, Shanghai, China; 5 CAS Center for Excellence in Brain Science and Intelligence Technology, Shanghai, China; 6 Shanghai Research Center for Brain Science and Brain-Inspired Intelligence, Shanghai, China

## Abstract

During the second World Summit of Human Gene Editing, Jiankui He presented the gene-editing project that led to the birth of two baby girls with man-made C-C chemokine receptor type 5 (*CCR5*) mutations. This extremely irresponsible behavior violated the ethical consensus of scientists all over the world. His presentation revealed a troubling lack not only of basic medical ethics but also of the requisite understanding of genetics and gene editing. Here, we review the rationale and design of his experiment along with the presented data, and provide our scientific criticism of this misconduct.

## Abstract 2

Last year, a gene-editing project led by Jiankui He resulted in the birth of two baby girls with engineered CCR5 mutations. In this Perspective article, two researchers working in the gene-editing field in China provide their scientific criticism of this misconduct.

On November 25, 2018, Jiankui He, an associate professor from Southern University of Science and Technology, announced that two babies with edited C-C chemokine receptor type 5 (*CCR5*) genes had been born in China. This genetic modification, he claimed, would render these babies immune to HIV infection. On November 28, He presented the experimental data of this project at the second World Summit of Human Gene Editing. While solid evidence of this experiment remains to be disclosed and the veracity of such claims ascertained, the experimental design and data presented at the summit revealed serious misconduct on both the scientific and ethical levels. As researchers working in the gene-editing field in China, we were completely shocked by this news. It would appear that He had been doing this work in secret. As far as we know, He has not published noteworthy scientific papers in the gene-editing field and was not actively involved in the gene editing community in China. We were enraged by this extremely irresponsible misconduct, which clearly violated the regulatory and medical ethics of China and nations all over the world. Here, we focus on the pitfalls of the scientific aspects, assuming the data He presented were true, because we believe that responsible scrutiny and discussion of this event requires a good understanding of the scientific facts.

First, we would like to criticize his overall rationale. He claimed that he edited the *CCR5* gene to prevent HIV infection in the babies, whose father is an HIV carrier (the mother does not carry the virus). Yet gene editing in embryos is completely unnecessary to prevent HIV transmission to the fetus. It is possible for an HIV-positive father to generate healthy babies using established Assisted Reproductive Technology (ART) with an extraordinarily high success rate [1]. As for considering future immunity to HIV infection, simply avoiding potential risk of HIV exposure suffices for most people. Therefore, editing early embryos does not provide benefits for the babies, while posing potentially serious risks on multiple fronts, which we will discuss next.

The *CCR5* gene encodes a receptor on white blood cells that HIV-1 uses, along with another receptor, to infect human cells. A naturally occurring *CCR5Δ32* allele is present in certain European populations. Although both heterozygous and homozygous individuals have slower progression or resistance to HIV infections [2, 3], even homozygous *CCR5Δ32* individuals can still be infected by certain HIV strains [4]. Individuals carrying the *CCR5Δ32* allele are in general healthy; however, this allele exists in very low frequency in non-European populations, and no homozygous mutant has been identified in Chinese populations [5, 6]. Therefore, it is very difficult to predict the risk of introducing the *CCR5Δ32* allele or other *CCR5* mutant alleles into a Chinese genetic background. While He claimed that there was a long-term health follow-up plan, there are no details on who will fund this or assume responsibility in the event that any medical issues arise.

Next, we’ll address his data. He first presented the data in *Ccr5* knockout (KO) mice in order to evaluate, “would loss of *CCR5* at the embryos stage by CRISPR/Cas9 gene editing cause undesirable genetic, physiological, or behavioral consequences?” (all contents in the quotation marks are quoted directly from He’s presentation slides). This is absurd. It is not possible to answer that question simply by comparing histology staining of four different tissues without any quantification and by doing two simple behavior tests in mice. The quality of the science is very poor and superficial. For example, the data from the novel object investigation behavior test suggested that there was a difference between the wild-type (WT) and *Ccr5* KO mice, although the *P* value is above 0.05. Further evaluation using a larger sample size is necessary before claiming *Ccr5* KO did not cause any behavioral phenotype. A cursory literature search would have revealed that *CCR5* has normal immune functions as a receptor of chemokines, and *CCR5* KO mice have natural killer cell (NK)-related phenotypes leading to higher risks for various viral infections [7–9].

Next, He designed multiple single-guide RNAs (sgRNAs) and tested their efficiency in human cell lines and monkey embryos. These are very routine procedures used for gene-editing experiments. After Clustered Regularly Interspaced Short Palindromic Repeats and the CRISPR-associated protein 9 (CRISPR-Cas9) components are delivered into cells, a DNA double-strand break (DSB) will be generated at the target genomic locus. Either the nonhomologous end joining (NHEJ) repair process or the homology-directed repair (HDR) pathway is employed to repair this DNA DSB. NHEJ repair often leads to small insertions or deletions (indels), while HDR results in perfect repair or precise genetic modification at the targeted site. He’s presentation showed only the characterization of indel mutation rates via NHEJ repair; no experiment designed to introduce the *CCR5*Δ32 allele via HDR repair was shown, suggesting that He had no intention of generating the *CCR5*Δ32 allele. As far as we know, *CCR5* mutant indel alleles other than the *CCR5*Δ32 allele do not exist in human populations at a high frequency. Previous studies suggested that expression and stability of the truncated CCR5Δ32 protein in *CCR5*^*−*/*−*^ individuals could also contribute to the HIV-resistance phenotype [10]. Therefore, other *CCR5* null alleles cannot simply be equated to the *CCR5*Δ32 allele when considering potential benefits and risks. Moreover, in-frame indel mutations could potentially generate gain-of-function mutations, the risks of which are even more difficult to predict.

He attempted to optimize the microinjection procedure using monkey zygotes, and performed sequencing to evaluate the gene-editing efficiency and level of mosaicism. Because his data have not been published on any platform as a research paper, the information shown on PowerPoint slides is insufficient for vetting. From what we can tell from his presentation, despite various attempts, mosaicism remains a problem in the monkey embryo experiments.

He then translated his microinjection protocol to human embryos. As illustrated by He’s data and previous studies, injecting embryos at the metaphase II (MII) stage and using Cas9 protein instead of Cas9 mRNA may reduce the mosaicism but does not eliminate it [11, 12]. Moreover, this strategy only works on NHEJ-mediated gene knockout, not on HDR-mediated precise gene repair. While many strategies for increasing HDR have been reported in cell lines [13], whether they are applicable in human embryos remains an open question. Work done by the Mitalipov’s group suggests that the maternal allele could serve as a template for gene repair to achieve correction of pathogenic mutation [12], but other groups have argued that Cas9 may induce large-scale deletions or rearrangements that lead to false positive results using PCR-based genotyping [14]. These scientific debates reveal our incomplete understanding of the DNA-repair mechanisms and outcomes associated with gene editing in human early embryos and suggest that He probably underestimated the rate of mosaicism and the risk of introducing harmful genetic alterations.

To assess mutations caused by off-target editing, He established one human embryonic stem cell (hESC) line from the edited human embryos. Here, again, the quality of the science is substandard. Only one hESC line was derived from one edited human embryo, which was then used for whole-genome sequencing (WGS) to detect potential off-target mutations. During the process of hESCs’ derivation and expansion, many genetic alterations will occur [15]. Therefore, to identify the true off-target mutations caused by gene editing, multiple hESC lines need to be established from edited and unedited embryos and characterized by deep sequencing and extensive bioinformatic analysis.

He further claimed that he performed so-called single cell–based WGS on preimplantation genetic diagnosis (PGD) samples from 19 edited human blastocysts to assess on-target and off-target editing events, before choosing the ones to transfer into recipients. Twelve out of nineteen embryos contained WT alleles, indicating the *CCR5* gene was not completely edited in these embryos. Importantly, there is no mature and reliable technique for single cell–based WGS to address the off-target mutations [16]. The whole genome amplification process, which amplifies the single copy of the genome to a large enough quantity for WGS, introduces many artificial mutations [16]. In addition, mosaicism is a major concern that cannot be addressed by PGD, as we cannot sequence all cells in an embryo [17]. This means that even if the tested cells are correctly edited, there is still a non-negligible risk that other cells in the embryo remain unedited or carry unwanted mutations that may have unpredictable consequences. Thus, He’s claim is unreliable.

In addition to potential off-target effects, it has been reported that DSBs generated by CRISPR-Cas9 may also lead to on-target mutagenesis effects [18, 19]. Besides the types of insertions, deletions, translocations, and rearrangements, on-target effects include large chromosome deletions, chromosome truncations, and homozygosis of the genome by inter-homology repair. Currently, no single method could detect all these types of off-target mutations, especially when they occur at a very low frequency.

After the two baby girls were born, He’s team collected DNA from their cord blood, umbilical cord, and placenta and performed WGS to confirm the success of *CCR5* editing. The WGS results suggested that only two different *CCR5* alleles existed in these samples, each one represented by approximately half of all sequencing reads. For Lulu, one allele remained WT, and the other allele had an in-frame deletion (−15 bp). For Nana, the two *CCR5* mutant alleles represented 100% of all sequencing reads at the *CCR5* target region, which suggests, quite surprisingly, that none of the mother’s tissue (containing WT *CCR5* allele) contaminated any of Nana’s samples. Yet, because the details of sample collection and data analysis are lacking, we cannot draw a robust conclusion. We strongly suggest that the authorities conduct a thorough examination of all the original data and disclose the facts to the scientific community and general public.

In conclusion, based on currently available information, we believe there is no sound scientific reason to perform this type of gene editing on the human germline, and that the behavior of He and his team represents a gross violation of both the Chinese regulations and the consensus reached by the international science community. We strongly condemn their actions as extremely irresponsible, both scientifically and ethically. We strongly urge the international community of scientists and regulators to initiate a comprehensive discussion as soon as possible to develop the criteria and standards for genome editing in the human germline for reproductive purposes. After reaching a clear consensus, clear and strict laws need to be passed, implemented, and enforced at an international level. We also believe, however, that it is necessary to further develop and improve the technologies for introducing precise genetic modifications into the human germline, including early embryos, sperm, and oocytes, using in vitro experimental setups. These improved technologies may provide solutions for genetic diseases—but only when consensus has been met and a regulatory framework has been put in place for treating specific medical implications.

## Abbreviations

ART: Assisted Reproductive Technology
CCR5: C-C chemokine receptor type 5
CRISPR-Cas9: clustered regularly interspaced short palindromic repeats and the CRISPR-associated protein 9
DSB: double-strand break
HDR: homology-directed repair
hESC: human embryonic stem cell
KO: knockout
MII: metaphase II
NHEJ: nonhomologous end joining
NK: natural killer cell
PGD: preimplantation genetic diagnosis
sgRNA: single guide RNA
WGS: whole-genome sequencing
WT: wild-type
